# Feeling Every Bit of Winter – Distributed Temperature Sensitivity in Vernalization

**DOI:** 10.3389/fpls.2021.628726

**Published:** 2021-01-27

**Authors:** Rea L. Antoniou-Kourounioti, Yusheng Zhao, Caroline Dean, Martin Howard

**Affiliations:** ^1^Computational and Systems Biology, John Innes Centre, Norwich Research Park, Norwich, United Kingdom; ^2^Cell and Developmental Biology, John Innes Centre, Norwich Research Park, Norwich, United Kingdom

**Keywords:** vernalization, temperature-sensing, mathematical modeling, FLC, Arabidopsis, climate change, temperature fluctuations

## Abstract

Temperature intrinsically influences all aspects of biochemical and biophysical processes. Organisms have therefore evolved strategies to buffer themselves against thermal perturbations. Many organisms also use temperature signals as cues to align behavior and development with certain seasons. These developmentally important thermosensory mechanisms have generally been studied in constant temperature conditions. However, environmental temperature is an inherently noisy signal, and it has been unclear how organisms reliably extract specific temperature cues from fluctuating temperature profiles. In this context, we discuss plant thermosensory responses, focusing on temperature sensing throughout vernalization in Arabidopsis. We highlight many different timescales of sensing, which has led to the proposal of a distributed thermosensing paradigm. Within this paradigm, we suggest a classification system for thermosensors. Finally, we focus on the longest timescale, which is most important for sensing winter, and examine the different mechanisms in which memory of cold exposure can be achieved.

## Introduction

Plants control their development in response to seasonal cues. A striking example of this is the floral bloom in spring. Monitoring seasons requires that plants read noisy signals over long time periods, as, for example, sampling temperature at a single time point cannot distinguish a cool period in autumn from the full length of winter. Different reproductive strategies differentially depend on seasonal monitoring. An overwintering, or winter annual habit, necessitates exposure to winter cold and restricts plants to one generation a year, but avoids summer mortality. In contrast, a rapid-cycling strategy without a cold requirement enables multiple generations each year if conditions allow (Satake, 2010). The overwintering requirement involves the process of vernalization, the acceleration of flowering by prolonged cold. The molecular basis of vernalization has been established in Arabidopsis, and is conserved throughout the Brassicaceae and in cereals (Dixon et al., 2019). In Arabidopsis, the central regulator is the flowering repressor *FLOWERING LOCUS C* (*FLC*), whose activity represses flowering in otherwise favorable conditions. *FLC* expression is repressed by cold and becomes epigenetically silenced (Michaels and Amasino, 1999; Sheldon et al., 1999). *FLC* is a repressor of the flowering promoter *FT*, so after a sufficiently long period of cold, this repression is released. *FT* itself also responds to temperature and daylength, so that with the long, warm days of spring, flowering is promoted.

**Figure 1:**
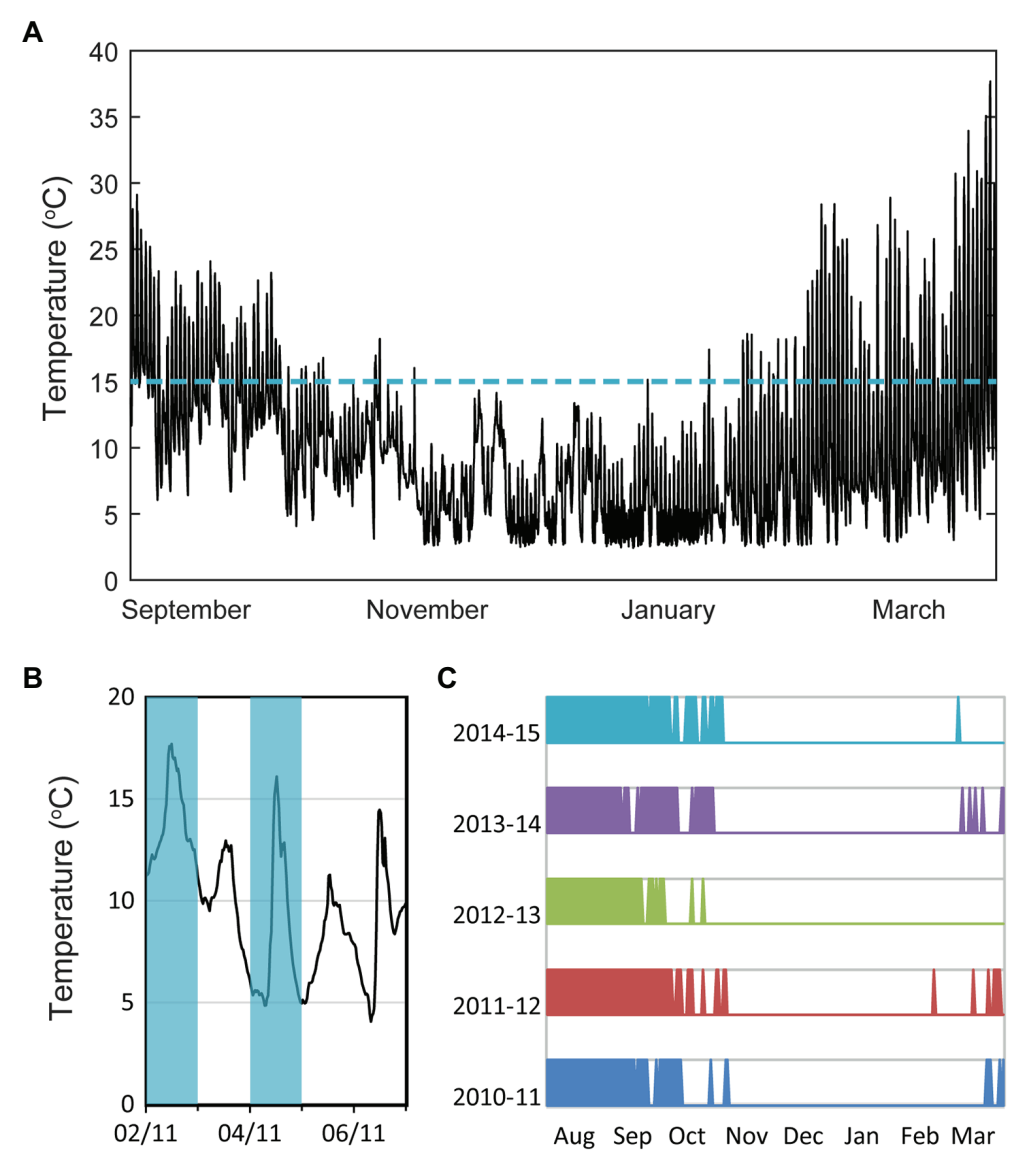
Importance of temperature fluctuations in plant seasonal sensing. **(A)** Temperature profile at experimental site in Norwich, United Kingdom, measured over 200 days from September 29, 2014 (Hepworth et al., 2018). **(B)** Schematic of classification of days according to whether the temperature fluctuated to above 15°C or not, during the day. This classification is used in the next panel. **(C)** Data from Norwich Airport, United Kingdom for the indicated years. Information provided by the National Meteorological Library and Archive – Met Office, United Kingdom, under the Open Government License (The National Archives, n.d.). Schematic shows coloration for days where the temperature fluctuated above 15°C, and white for other days, as indicated in **(B)**. The continuous white period matches winter.

Plants in the field experience complex temperature fluctuations and even over a single day these fluctuations can be as large as the variation between seasons (Figure 1A). Furthermore, seasons are variable each year, and a recent study has found that plants need longer vernalization times in regions where temperature correlations persist for longer periods (Zhao et al., 2020a), as this can lead to extended durations of unseasonable temperature. A combination of long-term temperature and short-term daylength information was found to give the best seasonal predictor. Therefore, the question of how plants sense temperature in natural conditions is of central importance, especially in times of a changing climate. In this review, we discuss this question in the context of vernalization, with a focus on the role of fluctuations and on sensing at multiple timescales, including long-term cold sensing. We concentrate on the winter sensing of the *FLC* gene and its upstream regulators in *Arabidopsis thaliana*.

## Dissecting Temperature Response in Natural Environments

Most knowledge of temperature sensing in plants comes from studies of plants grown under constant laboratory conditions. However, vernalization and seed dormancy in *Arabidopsis thaliana* were shown to be different in fluctuating vs. constant temperature conditions (Burghardt et al., 2016; Topham et al., 2017). Molecular analysis of the floral repressor locus, *FLC* and its regulator *VERNALIZATION INSENSITIVE3 (VIN3)*, showed that expression of neither gene responds to the average temperature, with the response instead more closely matching the extreme temperatures (Hepworth et al., 2018). In particular, *VIN3* expression is more strongly affected by the highest daily temperatures, while, independent of *VIN3*, *FLC* responds to the low temperature fluctuations. Further work (Antoniou-Kourounioti et al., 2018) showed that the night-time temperature was most important for *FLC* shutdown independently of VIN3, while *VIN3* itself responds similarly to day-time and night-time temperatures, despite having a diurnal pattern of expression (Hepworth et al., 2018).

These studies demonstrate the importance of temperature fluctuations in seasonal registration, a feature which will become more pertinent as climate variability increases. Predictions of plant responses (particularly crop yield) to climate change are important for breeding and policy decisions, as there is an expectation of a decrease in yield with warming (Liu et al., 2016). To accurately predict how plants will respond to new climate conditions, we need to understand the temperature features that are being sensed by the plant directly and how these are integrated.

Recently, field studies combined with mathematical modeling have effectively revealed the properties of the temperature sensing networks, thus giving insight into the underlying mechanisms. The use of field studies enabled environmental fluctuations to be properly incorporated, while the modeling helped dissect mechanisms too complex to discern by intuition alone. This approach with *A. thaliana* at three field sites showed that the *FLC* levels in natural autumns decrease slowly while the temperature is fluctuating to above 15°C daily (Hepworth et al., 2018). The rate of decrease was faster once the daily fluctuations in temperature did not reach 15°C. This feature is a surprisingly simple and reliable signature for onset of winter in Norwich, United Kingdom (Figures 1B,C) but will not be reliable in other regions or under climate change. Instead, a mathematical model based on the epigenetic mechanism that controls *FLC*, and the multiple temperature features identified (described in the next section), was developed for the *A. thaliana* Col *FRI* genotype. The model was able to reproduce the *FLC* expression pattern over the three field experiments (Antoniou-Kourounioti et al., 2018). In building this model, we were able to find the minimal temperature sensing network that can mimic the properties of the true network. Such a model can also make useful predictions about gene expression and flowering time responses under any potential temperature profile. Accordingly, the model was used to predict *FLC* expression under simple climate change scenarios. In addition, earlier work with field experiments in Japan working on the perennial *A. halleri* and using mathematical modeling, showed that the average temperature of the last 6 weeks best correlates with the *FLC* expression at any time of year (Aikawa et al., 2010). By integrating field data, mathematical modeling, and transplantation experiments, Nishio et al. uncovered a comprehensive H3K27me3-mediated chromatin regulation system at *A. halleri FLC* that is required for robust gene regulation in a fluctuating natural environment (Nishio et al., 2020a,b). Overall, a field study/modeling approach can provide a route for predicting phenological shifts, and thereby helping to develop robust crops in a future changing climate.

## Temperature Sensitivity at Different Timescales in a Distributed Thermosensing Paradigm

**Figure 2:**
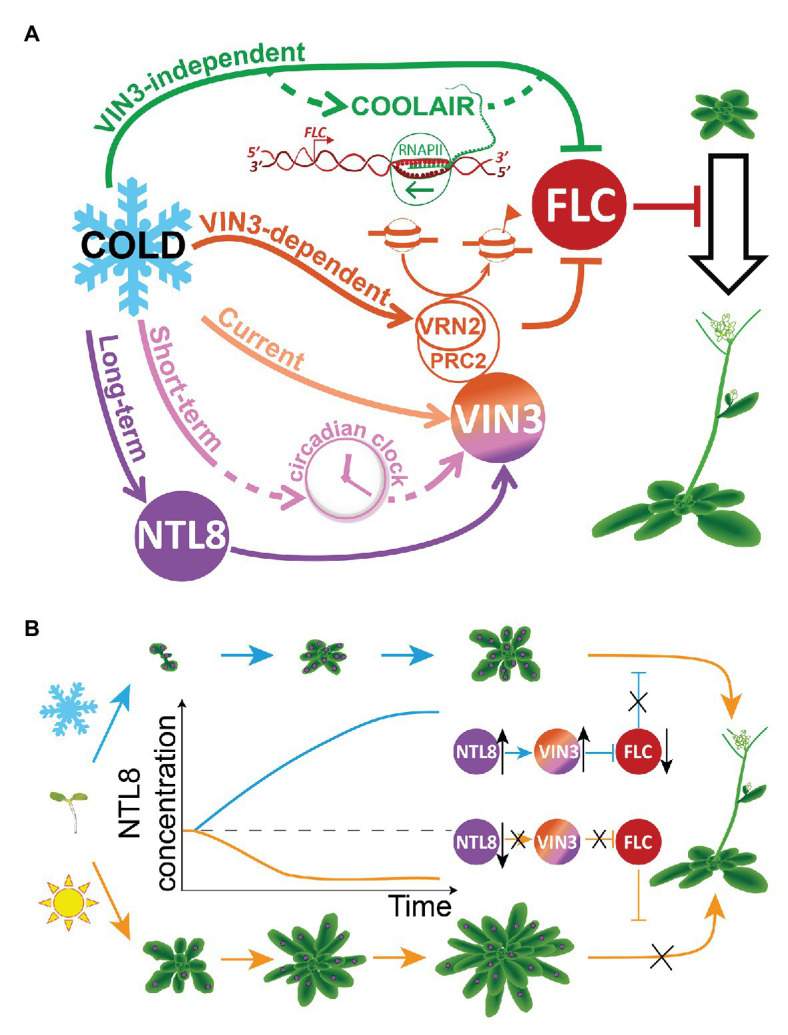
Distributed temperature sensitivities in the regulation of *FLOWERING LOCUS C* (*FLC*) and *VERNALIZATION INSENSITIVE3* (*VIN3*), and indirect long-term temperature sensing through NTL8 during vernalization. **(A)** Temperature sensitivities are widely distributed in the regulation of *VIN3* and *FLC* during vernalization. Cold regulates *VIN3* and *FLC* through multiple pathways: at least five separate pathways have been shown to be needed. In some cases, such as the “Long-term” pathway, the mechanism and components are known (NTL8; Zhao et al., 2020b). Known and postulated components are illustrated schematically in the diagram. **(B)** Temperature-dependent growth is indirectly exploited by the NTL8 protein to sense long-term cold. NTL8 concentration decreases when the plant grows fast in the warm (bottom, orange arrows/curves), but slowly increases when the plant grows slowly in the cold (top, blue arrows/curves). The slow accumulation of NTL8 protein holds the memory of cold exposure in the cold, allowing a slow increase in VIN3 concentration, which promotes the epigenetic repression of *FLC* – a repressor of the floral transition. Purple circles on the plant indicate NTL8 protein. The total amount of protein shown is the same in warm and cold, but the concentration is different due to the difference in growth rate.

In sensing seasonal information for vernalization, plants were found to integrate temperature information over multiple timescales in the expression of the *VIN3* and *FLC* genes (Antoniou-Kourounioti et al., 2018). *VIN3* is controlled by at least three separate temperature sensing elements (Antoniou-Kourounioti et al., 2018), where temperature information at the three timescales is integrated into the regulation of *VIN3* expression (Figure 2A). At the longest timescale (“Long-term”), *VIN3* expression is slowly increased over weeks (Sung and Amasino, 2004; Antoniou-Kourounioti et al., 2018; Hepworth et al., 2018). A regulator controlling this increase must be slowly accumulating and so cannot also respond quickly. At the shortest timescale (“Current”), the response occurs within 1–2 h, and would override any slow response (Hepworth et al., 2018). Therefore, the same property of a regulator cannot hold both temperature inputs (of current and long-term temperature). However, parallel properties (e.g., concentration and molecular activity) could in theory each hold part of the temperature information. The third, intermediate temperature timescale (“Short-term”) works over a night-and-day cycle, holding a short-term memory of recent temperature that is reset each evening, likely by the circadian clock (Antoniou-Kourounioti et al., 2018). Though the intuition described above can explain the fundamental timescale incompatibility, this issue was not obvious directly from experiments. Instead, mathematical models were developed with the aim to minimize the number of independent temperature inputs: this approach then demonstrated that multiple such inputs with different timescales were necessary.

*FLC* responds to temperature *via* the cold-induced VIN3, but also through a mechanism independent of VIN3, with a separate temperature input (Antoniou-Kourounioti et al., 2018). Similarly to *VIN3*, this mechanism is likely to involve multiple inputs, including through regulation of the *FLC* antisense transcript *COOLAIR* (Figure 2A). *COOLAIR* is cold-induced and antagonizes *FLC* expression (Swiezewski et al., 2009; Rosa et al., 2016). Furthermore, the VIN3-dependent epigenetic silencing of *FLC* has temperature sensitivity beyond the *VIN3* induction itself, as suggested by the absence of *FLC* silencing in lines expressing *VIN3* in the warm (Lee et al., 2015; Antoniou-Kourounioti et al., 2018). A candidate that could mediate this additional temperature sensitivity is VERNALIZATION2 (VRN2), a component of Polycomb Repressive Complex 2 (PRC2). VRN2 also accumulates in the cold (Wood et al., 2006) by a mechanism that involves inhibition of degradation through the Arg/N-end rule pathway (Gibbs et al., 2018). Overall, we can conclude that vernalization uses multiple temperature inputs to register the progression of winter, suggesting that multiple thermosensors are involved (Figure 2A).

This multiplicity of inputs required to control a single process suggested the principle of distributed thermosensing, by which the inherent temperature sensitivity of multiple molecules and reactions is combined, rather than only through specialized thermosensors (Antoniou-Kourounioti et al., 2018). The latter would require that multiple specialized sensors evolved to control each temperature responsive process, with temperature compensation used to ensure no response from many otherwise naturally temperature sensitive molecules or reactions. Given that temperature compensation is probably a difficult response to generate, utilizing the widespread but weaker temperature sensitivity of many elements in distributed thermosensing is likely to be an easier strategy to evolve, as well as being highly redundant. By definition, distributed thermosensing allows a combination of many small responses to be integrated and so gives more room for a precise response to a large range of temperature stimuli. This flexibility may be needed for plants in the case of vernalization or that of thermomorphogenesis, the effect of ambient warm temperatures on plant morphogenesis, another process where multiple temperature sensors have been discovered (Jung et al., 2016; Legris et al., 2016; Chung et al., 2020; Jung et al., 2020).

## Classification of Thermosensors

What kinds of thermosensor are possible in the distributed sensing paradigm? We suggest a classification into “direct” or “indirect” sensors, and within these two categories, a spectrum of sensitivity to temperature. In the “direct” case, there can be molecules with a strong, switch-like response to temperature changes, e.g., RNA thermoswitch (Chung et al., 2020) or the formation of speckles of ELF3 at 35°C due to its prion domain (Jung et al., 2020). These strong thermosensors are powerful, but not necessarily representative and it is important to realize that they are necessarily part of a larger network, which will itself also be thermosensitive. Molecules with weakly temperature sensitive properties are therefore likely to be pervasive within a distributed thermosensing paradigm. These could be combined to give a synergistic response much stronger than the sum of its individual parts, giving a strongly temperature sensitive network.

In contrast, “indirect” sensors are qualitatively different: the temperature sensitivity is not in the “sensor” molecule, but instead non-temperature-sensitive properties of this molecule allow it to couple to the temperature sensitivity of a separate process. Rather than simply responding to this separate temperature input, indirect sensors can use it to measure a new feature of temperature, thus creating a temperature response that was not available in the input signal. Below, we will describe an example of this type of sensor involving NTL8, *VIN3*, and *FLC* (Zhao et al., 2020b). Stability and tissue localization allow NTL8 to use the temperature sensitivity of growth (which responds to recent temperature) to create a new temperature-sensing mechanism that measures temperature at a different timescale, in this case, long-term cold exposure duration.

Distributed sensing suggests that all of these sensor types can potentially be important for the plant to respond to temperature. Understanding how direct and indirect sensors, both strong and weak, are combined in the context of vernalization could therefore be valuable for a better understanding of temperature sensing in general.

## Indirect Long-Term Cold Sensing Through Temperature-Dependent Growth

As described above, *VIN3* exhibits a long-term response to the duration of cold exposure (Antoniou-Kourounioti et al., 2018; Hepworth et al., 2018). This response was found to be graded at the single cell level, with *VIN3* expression increasing over time in each cell in the cold (Antoniou-Kourounioti et al., 2018). To understand the genetic basis of the temperature inputs of *VIN3* regulation during vernalization, a genetic screen was performed, and mutants with unusually high levels of *VIN3* expression in warm conditions were identified (Zhao et al., 2020b). These plants carry dominant mutations in the gene *NTL8* or its homolog *NTL14*, which encode proteins that directly regulate gene expression. In these mutants, NTL8 and NTL14 are more active than in the wildtype and so the mutant plants bypass the requirement of prolonged cold exposure to increase *VIN3* expression. Furthermore, in plants where both *NTL8* and *NTL14* are absent, *VIN3* expression in the cold is attenuated.

In the wildtype case, the transcript level of *NTL8* does not change over time in the cold. However, NTL8 protein concentration gradually increases. Normally, the timescale of any protein dynamics is dictated by its degradation rate. Indeed, NTL8 is quite stable, in keeping with its slow response. This protein stability is observed both in warm and cold conditions, arguing against a hypothesis whereby slow NTL8 accumulation is due to an enhanced stability of NTL8 in the cold. To help elucidate the underlying mechanism of accumulation, a computational simulation was generated to explore the effect of growth on the NTL8 protein dynamics. In warm conditions, fast growth led to the fast dilution of the NTL8 protein concentration, while slow growth in cold conditions led to slow dilution, and thus a slow, gradual increase of NTL8 protein concentration (Figure 2B). The mathematical model showed that slow growth alone is sufficient to drive an increase in NTL8 concentration in the cold. A clear prediction of this hypothesis was that inhibiting growth in the warm would also cause NTL8 accumulation, and this was subsequently observed experimentally. Overall, through a combination of experimental and theoretical approaches, it was demonstrated that NTL8 measures the duration of the cold at least in part through reduced dilution due to cold-inhibited growth.

We can now see why the NTL8 mechanism is an example of “indirect” sensing. Here, the long lifetime of NTL8, a property seemingly unrelated to thermosensing, is an essential requirement. This long lifetime allows NTL8 to couple to the temperature-dependent growth dynamics of the plant. Note that growth responds rapidly to temperature, yet NTL8 can respond at a much longer timescale due to its long lifetime. This allows its concentration to integrate over the plant’s temperature-dependent growth history. NTL8 then transcriptionally activates *VIN3* which inputs long-term cold information into *FLC* epigenetic repression. Interestingly, the long-term cold information held in trans in the NTL8 cellular concentration is in a quantitative/graded form. This is in contrast to the cell-autonomous, ON/OFF digital epigenetic repression of *FLC*, mediated by histone modifications, which holds memory of the cold in cis after the cold has passed (Angel et al., 2011; Berry et al., 2015). These different memory systems reflect the different biophysical constraints faced by the system in the cold and warm. In the warm, growth would rapidly compromise any cold information held in a graded concentration, due to dilution. Hence, the cold duration information must be copied from the NTL8/VIN3 module to an alternative form, stable to growth, and also to DNA replication and division. This is achieved by conversion to an ON/OFF digital cis memory format encoded at *FLC* chromatin through histone modifications.

## Discussion

How plants integrate daily and seasonal fluctuating temperatures to maximize their fitness and survival has been a long-standing fundamental question. Recent advances in temperature sensing have revealed key features of how vernalization proceeds in the field, highlighting the importance of performing research in field conditions, or controlled fluctuating conditions in the laboratory. Such work, in combination with mathematical modeling, has uncovered a multiplicity of thermosensors and fostered the concept of distributed temperature sensing. Working within this paradigm, and our proposed thermosensor classification, will allow researchers to identify new types of sensing mechanisms with a focus on the overall thermosensory network.

Climate warming will massively affect the phenology of many plants, including major crops. Knowledge of thermosensing will be instrumental in breeding weather-proof crops in order to combat climate change. One interesting direction will be to see how the multiplicity of sensors due to the distributed thermosensing principle will be implemented in polyploid species, where multiplicity also comes from the many gene copies.

## Author Contributions

All authors listed have made a substantial, direct and intellectual contribution to the work, and approved it for publication.

### Conflict of Interest

The authors declare that the research was conducted in the absence of any commercial or financial relationships that could be construed as a potential conflict of interest.
